# SF-6D utility scores of smokers and ex-smokers with or without respiratory symptoms attending primary care clinics

**DOI:** 10.1186/s12955-019-1115-z

**Published:** 2019-03-15

**Authors:** Sau-nga Fu, Man-Chi Dao, Carlos King-Ho Wong, Wai-cho Yu

**Affiliations:** 1 Department of Family Medicine & Primary Health Care, Kowloon West Cluster, Hospital Authority, Hong Kong Special Administration Region, China; 2 Department of Family Medicine & Primary Care, Li Ka Shing Faculty of Medicine, University of Hong Kong, Hong Kong Special Administration Region, China; 3 Department of Medicine and Geriatrics, Princess Margaret Hospital, Hong Kong Special Administration Region, China; 4 Present Address: G/F, Ha Kwai Chung General Outpatient Department, 77 Lai Cho Road, Kwai Chung, N.T., Hong Kong Special Administration Region, China

**Keywords:** Smoking, Primary Care, Health-Related Quality of Life (HRQoL), Spirometry, Respiratory Symptoms, SF-6D, BCSS

## Abstract

**Introduction:**

The aim of this paper is to find out generic preference-based Short-Form 6 Dimensions (SF-6D) utility scores of smokers and ex-smokers with varying cigarette exposure, with and without respiratory symptoms.

**Methods:**

Seven hundred thirty one people aged ≥30 with a history of smoking who attended 5 public primary care clinics completed a cross-sectional survey using SF-6D utility score, Breathlessness, Cough, and Sputum Scale (BCSS©) and office spirometry.

**Results:**

Most of the subjects were men (92.5%) in an older age group (mean age 62.2 ± 11.7 years). About half of them (48.3%) were current smokers while the other half (51.7%) were ex-smokers. More than half of them (54.2%) reported mild respiratory symptoms (mean BCSS score 0.95 ± 1.12). The most common symptoms were sputum (45.1%), followed by cough (34.2%) and breathlessness (6.0%). The SF-6D overall utility score was 0.850 ± 0.106. The subjects reported significantly lower SF-6D scores when they had breathlessness (0.752 ± 0.138; p = < 0.001), cough (0.836 ± 0.107; *p* = 0.007), sputum (0.838 ± 0.115; *p* = 0.004) or any of the above symptom (0.837 ± 0.113; *p* < 0.001). In both groups of current smokers and ex-smokers, there was no statistically significant difference in the scores among light, moderate or heavy smokers. In the Tobit regression model of factors affecting SF-6D utility score, subjects who reported more respiratory symptoms (i.e. higher BCSS©) had lower SF-6D scores (B = − 0.018 ± 0.007, *p* < 0.001), while men had higher SF-6D scores than women (B = 0.037 ± 0.031, *p* = 0.019). Subjects who attended middle or high school had higher SF-6D score than those attended the University or above. The presence of airflow obstruction was not associated with the score.

**Conclusions:**

The study yielded SF-6D utility scores of smokers and ex-smokers with different reported cigarette exposure, which could be useful in future clinical studies and cost-effectiveness analysis.

## Introduction

Recent systematic review demonstrated overall negative association of Health-Related Quality of Life (HRQoL) with smoking [1]. Current smokers usually reported lower HRQoL than ex-smokers or non-smokers. Heavy smokers often reported lower HRQoL scores than medium or light smokers [2, 3]. Longitudinal studies often showed an improvement of HRQoL after smoking cessation [4]. The HRQoL scores of both male and female ex-smokers were found to be improved between 2 and 5 years after quitting [5]. Status of smoking, gender and level of education were shown to be associated with HRQoL scores across different populations worldwide with different socioeconomic background [6, 7].

Individuals with more severe clinical symptoms often reported a lower HRQoL utility score. Patients with more respiratory symptoms consumed more health care resources [6], had poorer clinical outcomes, such as a decline in pulmonary functions and development of chronic obstructive pulmonary disease (COPD) [7] and a subsequently impaired HRQoL [8]. The link between smoking and respiratory symptoms was clearly demonstrated in a review study [9]. Greater tobacco exposure was associated with a higher risk of developing symptoms of chronic bronchitis, i.e. chronic cough and phlegm [10], and developing exacerbations with acute respiratory symptoms [11]. Smoking cessation leads to improvement of respiratory symptoms [2].

The relationships between respiratory symptoms, airway abnormality by spirometry and impaired HRQoL were also demonstrated in a National US survey [12]. Subjects from two large multi-centre cohorts in Europe reported a significantly lower Medical Observation Survey Short-Form 36 (SF-36) physical component score if their spirometry results demonstrated either restrictive or obstructive patterns as compared to those within normal ranges [13]. It is important for clinicians to understand the correlations between smoking status, level of cigarette exposure, respiratory symptoms, any airflow limitation and the HRQoL score as a clinical outcome.

Previous studies of smokers have commonly applied SF-36 or SF-12 as a generic outcome measurement of HRQoL in different populations. There were limited studies on the preference-based measures of health for subjects with a history of smoking using the Short Form 6 Dimension (SF-6D) utility score [1]. The SF-6D utility scores of older subjects attended lower educational level, which characterized our study population, were not worked out before. Therefore, this study aimed to find out the SF-6D utility score of smokers and ex-smokers attending primary care clinics. The calculated utility scores will be useful for future health economic evaluations. Based on previous observed association between the amount of daily cigarette smoking, sociodemographic variables and HRQoL score [5, 8, 14, 15], we hypothesized that among patients attending primary care clinics, ex-smokers might have higher SF-6D utility score when compared with current smokers. Heavy smokers might have lower utility scores when compared with moderate smokers or light smokers whether or not they have given up smoking. Finally, patients with more respiratory symptoms or airway obstruction demonstrated by spirometry might have lower utility scores when compared with patients with fewer respiratory symptoms or with no airway obstruction.

## Materials and methods

The study protocol was approved by the Kowloon West Cluster Research Ethics Committee, Hospital Authority of Hong Kong, and followed the principles of the Declaration of Helsinki [16]. All participants provided written informed consent for data collection. This study was performed in five public primary care community clinics in the Kwai Chung and Tsing Yi Districts of Hong Kong from April to July of 2014. The study clinics mainly provide primary health care services to the older people from the lower socioeconomic class. They are government funded outpatient clinics which provide doctors and nurses consultation services, including smoking cessation counselling service.

### Study subjects

During the study period, all attending patients’ computerized medical records were screened by clinic nurses on the day of consultation. All “smokers” or “ex-smokers” were potential subjects, regardless of their reasons for consultation. Clinic nurses checked potential subjects’ eligibility according to the recruitment criteria by confirming their history of smoking and any known diagnosis of chronic respiratory diseases. Informed consents were then obtained. Trained research nurses subsequently conducted face-to-face interviews using structured questionnaires and performed office spirometry on the subjects.

### Inclusion & exclusion criteria

All subjects had to be aged 30 or above and have a history of smoking (i.e. persons who reported smoking at least 100 cigarettes during their lifetime, according to the Centre of Disease Control & Prevention criteria 2011) [17]. Eligible subjects answered “yes” to the question: “Have you ever smoked more than 100 cigarettes in your whole lifetime?” [18], and they answered “no” to the question “Do you have any chronic lung disease which required you to see doctors regularly?” Subjects who were unable to perform spirometry, had previous diagnoses of chronic lung diseases including asthma, chronic obstructive pulmonary disease (COPD), pulmonary fibrosis, bronchiectasis and restrictive lung disease were excluded from the study. Pregnant women were also excluded from the study.

### Basic data collection

Research nurses interviewed participants using standard questionnaires in Cantonese (the local Chinese dialect) to collect the following data:

1. Demographics: gender, age, occupation
2. Smoking status, including intensity and duration, by using the World Health Organization and Centers for Disease Control and Prevention Global Adult Tobacco Survey (GATS).
3. Known chronic illnesses according to subjects’ medical records.
4. Assessment of acute respiratory symptoms by the validated Chinese version of Breathlessness, Cough, and Sputum Scale (BCSS, copyright 2003), was used with permission from the AstraZeneca Company [19].
5. Assessment of preference-based utility score by the SF-6D [20].

### Tools

GATS is a tool used in the Global Tobacco Surveillance System which provides a consistent framework for surveillance of adult smoking habit. It includes standard sampling procedures and core questionnaire items [17]. We adopted the three basic questions that measure tobacco smoking prevalence. The first is on current tobacco smoking status, the second on past daily smoking status, and the third is on the quantity of tobacco products smoked per day. Subjects are defined as current smokers if they are still smoking, and as ex-smokers if they are not currently smoking but have smoked in the past. The quantity of tobacco products smoked per day was defined by the question, “On average, how many of the following products do you currently smoke each (day/week)?” Six specific types of tobacco products were listed, plus one “any other” option. All cigarette smokers or ex-smokers were grouped according to their daily consumption. A light smoker is defined as one who smokes 1 to 9 cigarettes per day, while a moderate smoker smokes 10 to 19 cigarettes per day, and a heavy smoker smokes 20 cigarettes or more per day. We also calculated the number of pack-year from the reported number of cigarettes per day and the duration of smoking.

The BCSS is a three-item patient-reported score of the presence of three common COPD symptoms: breathlessness, cough and sputum. Subjects were asked to evaluate each symptom on a 5-point Likert scale, ranging from 0 to 4, with higher scores indicating more severe manifestation of symptoms. The total score is expressed as 0–12. The tool has been validated to assess daily respiratory symptoms in COPD patients [21].

The SF-6D is a preference-based measure of health with a six-dimensional health status classification: physical functioning, role functioning, social functioning, pain and discomfort, mental health and vitality [22]. It was derived from the SF-36 Health Survey, a widely used generic health profile developed in the US [23]. The subjects select one of the levels (up to level 4 or level 6) in each dimension which best describes their current health status. For example, in the dimension of physical functioning, the level 1 statement is: “Your health does not limit you in vigorous activities”, while the level 6 statement is “Your health limits you a lot in bathing and dressing.” The scoring algorithm of preference-based values in different levels (SF-6D Hong Kong Value Set) were derived and locally validated from 126 subjects from a primary care practice in Hong Kong [24, 25]. The total SF-6D value is the summation of 6 dimensions scoring. The theoretical plausible ranges start from 0.315 (the worse possible health state) to 1 (full health), according to the Chinese Hong Kong population specific scoring algorithm. The single value can be used to construct quality-adjusted life years (QALYs) in health economic evaluations. The authors have registered in the University of Sheffield website [26]. The approval for use under license was sought.

Spirometry was done according to the American Thoracic Society criteria by trained research nurses using a Spirolab III® spirometer (Medical International Research USA, Inc.) with subjects in a seated position [27]. The spirometric reference values for Hong Kong adults according to Ip et al. (2006) were adopted [28]. Airway obstruction was defined as a value less than 0.7 for forced expiratory volume in one second (FEV1) over forced vital capacity (FVC).

### Statistical analysis

The primary study outcome is the overall SF-6D utility score in patients with a history of smoking in primary care setting. The theoretical utility scores range from 0.315 to 1 using the Hong Kong population score [25]. The median and interquartile range of the overall SF-6D utility score was reported. Subgroup mean SF-6D utility scores were calculated for different sociodemographic groups, the quantity of cigarette smoked per day, the presence of respiratory symptoms and whether they had airway obstruction. Independent t-test and ANOVA test were used to compare the difference in scores for subgroup analysis. Normal distribution of the overall score was tested by using the Kolmogorov-Smirnov Test.

Based on our hypothesis, we examined the effects of sociodemographic and clinical factors on SF-6D utility score by the Tobit regression model. The Tobit regression model was applied because of the continuous features of a skewed response with a moderate ceiling effect of the SF-6D scores [29]. The coefficient, and its 95% confidence intervals were estimated. All significance tests were two-tailed, and a *P*-value of less than 0.05 was considered significant. Statistical analyses were performed using STATA version 13.0 (College Station, TX: StataCorp LP).

## Results

Of the 770 eligible subjects who were approached, 16 refused to participate in the study. The response rate was 97.9%. Twenty-three subjects were excluded because of pregnancy (*n* = 1), having kyphoscoliosis (*n* = 2), being physically unfit to blow (*n* = 11) or having known chronic lung disease (*n* = 9). A total of 731 subjects completed the questionnaires and the spirometry test. The subjects’ sociodemographic and clinical characteristics are shown in Table 1. Most of the subjects were men (92.5%) in the older age group (mean age 62.2 years, SD = 11.7). The majority (73.1%) had only attended middle school or below. One-third (32.4%) were actively employed. Many subjects were either overweight (24.1%) or obese (47.1%) according to the World Health Organization Asian body mass index (BMI) cut-off level [30]. More than 70% of them had chronic illnesses: two-third had hypertension and one-third had diabetes mellitus.

**Table 1:** Sociodemographic and Clinical Characteristics of 731 smokers and ex-smokers

Characteristics	Total (*N* = 731)	Characteristics	Total (N = 731)
*Sociodemographic*		*Clinical*	
Age (year; mean ± SD)	62.2 ± 11.7	Body weight (kg; mean ± SD)	66.9 ± 11.4
Gender (%)		Body height (m; mean ± SD)	1.6 ± 0.1
Male	676 (92.5%)	BMI (kg/m^2^; mean ± SD)	25.0 ± 3.6
Female	55 (7.5%)	BMI cutoff	
*Education*		Underweight (< 18.5)	14 (1.9%)
Primary or below	338 (46.2%)	Normal (≥18.50 - < 23)	197 (26.9%)
Middle School	197 (26.9%)	Overweight (≥23 - < 25)	176 (24.1%)
High School	153 (20.9%)	Obese (≥25)	344 (47.1%)
University or above	43 (5.9%)	Hypertension (%)	453 (62.0%)
*Occupation*		Diabetes (%)	242 (33.1%)
Retired	369 (50.5%)	Ischemic Heart Disease (%)	38 (5.2%)
Working	237 (32.4%)	Stroke (%)	43 (5.9%)
Others or unknown	125 (17.1%)	Spinal deformity (%)	3 (0.4%)
*Smoking Status*		Chronic pain for at least 6 months (%)	4 (0.5%)
Total Current smoker	353 (48.3%)	Other heart disease (%)	14 (1.9%)
• Light smoker	101 (28.6%)	Undertaken Heart or Lung Surgery (%)	2 (0.3%)
• Moderate smoker	147 (41.6%)	Pulmonary tuberculosis (%)	3 (0.4%)
• Heavy smoker	105 (29.7%)		
Total Ex-smoker	378 (51.7%)	Other lung disease (%)	2 (0.3%)
• Light ex-smoker	52 (13.8%)	Any Chronic illness (%)	537 (73.5%)
• Moderate ex-smoker	109 (28.8%)	Number of chronic illnesses (mean ± SD)	1.08 ± 0.84
• Heavy ex-smoker	212 (56.1%)		
Years since first smoking (year; mean ± SD)	42.9 ± 12.9		
Pack-year (mean ± SD)	34.2 ± 25.1		

*BMI* body mass index, *COPD* chronic obstructive pulmonary disease, *cpd* cigarettes per day

Regarding the subjects’ smoking history, about half (48.3%) of them were current smokers. All were cigarette smokers except one who smoked bamboo pipes. The mean number of years since first smoking was 42.9 years (S.D. = 12.9). The mean pack-year was 34.2 (S.D. = 25.1). About 21% of the subjects were light smokers (0–9 cigarettes per day), 35% were moderate smokers (10–19 cigarettes per day) and 43.4% were heavy smokers (20 cigarettes per day or more). More than half (56.1%) of the ex-smokers were heavy smokers, and less than 30% were moderate smokers. Current smokers reported fewer number of cigarettes per day when comparing with Ex-smoker. Less than 30% were heavy ex-smokers or light ex-smoker, more than 40% of them were moderate ex-smokers.

The BCSS score results are shown in Table 2 and Table 3. The mean BCSS score was 0.95 (S.D. = 1.12). 335 subjects (45.8%) had no acute respiratory symptoms (BCSS score 0) while 381 (52.1%) had BCSS score 1–3. The most prevalent symptom was having sputum (45.1%), followed by cough (34.2%) and breathlessness (6.0%).

**Table 2:** Breathlessness, cough and sputum score of the 731 study subjects

	Mean ± SD	Observed range
Total BCSS score = 0 (no symptoms) to 12 (all severe symptoms)	0.947 ± 1.121	0–9
Breathlessness (0–4)	0.071 ± 0.297	0–2
Cough (0–4)	0.378 ± 0.571	0–3
Sputum (0–4)	0.498 ± 0.598	0–4

**Table 3:** SF-6D utility scores of smokers, ex-smokers, and subjects with or without respiratory symptoms

Characteristics	N (%)	SF-6D score (mean ± S.D.)	Observed range / *P*-value*
Overall	731	0.850 ± 0.106	0.500–1
Pulmonary Symptoms			
Breathlessness			< 0.001
No symptom (0 point)	687	0.856 ± 0.100	
Have breathlessness (≥1 point)	44 (6.0%)	0.752 ± 0.138	
Cough			0.007
No symptom (0 point)	481	0.858 ± 0.104	
Have cough (≥1 point)	250 (34.2%)	0.836 ± 0.107	
Sputum			0.004
No symptom (0 point)	401	0.860 ± 0.097	
Have sputum (≥1 point)	330 (45.1%)	0.838 ± 0.115	
Total BCSS score			< 0.001
No symptom (0 point)	335	0.865 ± 0.094	
Any of the above symptom (≥1 point)	396 (54.2%)	0.837 ± 0.113	
Smoking status			0.892
Current smoker	353	0.849 ± 0.107	
Ex-smoker	378	0.851 ± 0.104	
Smoking status for current smoker			0.700
Light (< 10 cigarettes per day)	101	0.851 ± 0.106	
Moderate (10–19 cigarettes per day)	147	0.844 ± 0.109	
Heavy (≥20 cigarettes per day)	105	0.855 ± 0.107	
Smoking status for ex-smoker			0.360
Light (< 10 cigarettes per day)	52	0.867 ± 0.098	
Moderate (10–19 cigarettes per day)	109	0.854 ± 0.099	
Heavy (≥20 cigarettes per day)	212	0.845 ± 0.109	

*BCSS* Breathlessness, Cough, and Sputum Scale

*P-value of independent t-test / ANOVA test of SF-6D scores between patients with and without symptoms

The SF-6D utility scores in subjects with or without respiratory symptoms, in current and past smokers and in light, moderate and heavy smokers are shown in Table 3. The median overall SF-6D (Hong Kong) utility score was 0.87 and the interquartile range was 0.10. The utility score ranged from 0.5 to 1.0 (perfect health). The most commonly reported limitation in SF-6D domains was vitality (77.6%), followed by physical functioning (65.5%) and bodily pain (53.4%). Kolmogorov-Smirnov Test indicated that the SF-6D scores did not follow a normal distribution D (731) = 0.146, *p* < 0.001.

Table 4 shows the sociodemographic and clinical factors associated with the overall SF-6D utility score. In the Tobit regression model using the overall SF-6D utility score as a dependent variable, being male (coefficient = 0.037 ± 0.31, *p* = 0.019) was positively associated with higher scores. Subjects attended middle school (coefficient = 0.037 ± 0.35, p 0.039) and high school (coefficient = 0.039 ± 0.037, p 0.037) had significantly higher scores than those who attended the University or above. There was no significant difference in the SF-6D utility scores between subjects attended primary school or below and subjects attended university or above. Subjects who reported any respiratory symptoms in BCSS had lower SF-6D utility scores (0.836, S.D. = 0.113) than those without respiratory symptoms (0.865, S.D. = 0.094) (*p* < 0.001). A higher BCSS score (coefficient = − 0.018 ± 0.007, p < 0.001) was associated with a lower SF-6D utility score. Being a current smoker, an ex-smoker, the quantity of cigarettes smoked daily, or the number of years since first smoking had no significant effect on the SF-6D utility score. Other factors such as the presence of airflow obstruction (defined as FEV1/FVC < 0.7), age, education, occupation, body mass index and the number of chronic disease documented were also not associated with SF-6D utility score.

**Table 4:** Tobit regression model of Factors associated with overall SF-6D utility score

Factor	Coefficient	95% CI	*P*-value
Sociodemographic			
Age in year^a^	−0.001	(− 0.002,0.001)	0.289
Male	**0.037**	**(0.006,0.069)**	**0.019**
Education (University or above)			
Primary or below	0.022	(−0.012,0.057)	0.206
Middle School	0.037	(0.002,0.073)	0.039
High School	0.039	(0.002,0.075)	0.037
Occupation (Others)			
Retired	−0.006	(−0.035,0.022)	0.665
Working	0.018	(−0.007,0.042)	0.153
Current smoker	−0.006	(−0.023,0.012)	0.516
Years since first smoking	−0.001	(−0.002,0.001)	0.348
Cigarettes per day (Heavy)			
Light	0.013	(−0.009,0.034)	0.257
Moderate	0.001	(−0.017,0.019)	0.936
Clinical			
Underweight or normal, BMI < 23 kg/m^2^	−0.003	(−0.021,0.014)	0.718
Number of chronic diseases	0.004	(−0.006,0.014)	0.450
Pulmonary Symptoms in BCSS score^a^	**− 0.018**	**(−0.025,-0.011)**	**< 0.001**
Airway obstruction	0.010	(−0.013,0.032)	0.393

*BMI* body mass index, *BCSS* Breathlessness, Cough, and Sputum Scale, *COPD* chronic obstructive pulmonary disease, *CI* confidence interval

^a^Negative coefficients indicated the increase in one unit of independent variable was associated with decrease in SF-6D score

## Discussion

This is the first study illustrating the SF-6D utility score of primary care patients with a history of smoking. The majority of subjects were men (92.5%) who were relatively older (mean age 62.2) with lower educational level (less than 6% attended university or above). The prevalence of male subjects in our study may be explained by the fact that 83.3% of smokers in Hong Kong were men. This is further substantiated by local statistics in 2015 which reported the percentages of male and female daily cigarette smokers to be 18.6 and 3.2% respectively [31]. In addition, the World Health Organization Global statistics revealed that 36.1% of men and 6.8% of women globally were daily smokers [32]. Therefore the SF-6D utility scores calculated in this study might thus be applicable to other population with similar characteristics.

The resulting SF-6D utility score is essential for the calculation of QALYs in health economic evaluations. Since no similar investigation had been previously conducted, the utility scores from this study may serve as a particularly useful reference for future research on a predominantly male subject population who have a history of smoking in a primary care setting [1]. For instance, the median overall SF-6D utility score was 0.87 (observed range 0.5 to 1.0) obtained from the present study was similar to the SF-6D utility score measured in type 2 diabetes patients in Hong Kong primary care clinics (0.847, S.D. = 0.148) [26]. Another recent published study illustrated that the utility scores may be decreased to 0.745 for those developing chronic lung disease [33]. Such findings provide substantiated grounds for clinicians to explain to smokers the possibility of decline in HRQoL with development of chronic lung disease, so that early smoking cessation may prevent decrease in HRQoL.

We attempted to measure the association of HRQoL and smoking status, number of cigarettes smoked, patients’ gender and educational status. Similar to a previous study [5], we were unable to demonstrate the effect of the quantity of cigarette smoked (being light, medium or heavy smoker), being a current smoker or an ex-smoker, and the presence of airway obstruction on the SF-6D values in both the current smoker and the ex-smoker groups. This could be because our subjects were relatively older (mean age 62.2). More than half of them had already given up smoking.

The slightly higher SF-6D scores observed in male when compared to female subjects in this study was not demonstrated in previous studies [12, 29]. In a recent study of HRQoL among smokers, the scores were significantly different among women, but not in men [15]. In a study of subjects with chronic illnesses, male subjects had a significantly higher mental component score when compared with female subjects in the 40–59 age group. There were no differences in the SF-12 physical component score or mental component score between female and male subjects in other age groups [30]. It would be useful for future studies to address this possible difference.

In this study, the SF-6D utility scores varied between groups attended different educational levels. Association of educational level and HRQoL was also found in previous studies [15]. Charafeddine et al. found subjects attended higer educational level reported higher HRQoL when compared with those attended lower educational level. But the association pattern was different in this study. Subjects attended middle school or high school in this study reported higher SF-6D utility scores than those attended university or above. We found subjects who had attended primary school had no statistical significance in SF-6D utility scores when compared with those attended University or above.

Concerning the association between respiratory symptoms and the HRQoL score, this study found when subjects had either cough, sputum or breathlessness, they had lower SF-6D utility scores, which was the lowest when subjects reported breathlessness. It was agreed with the result of the United States National Health and Nutrition Examination Survey: it found that younger subjects (age 20–44) with any respiratory symptoms were consistently associated with impaired quality of life. In this study, having breathlessness was the least commonly reported symptoms. This finding aligned with another review study, the prevalence of breathlessness among subjects with a history of smoking might be variable (2–41%) [9]. The lowest observed SF-6D utility scores in subjects reported breathlessness may be explained by impairment in physical component of SF-36 shown in a previous study [12].

### Study limitations

This study explored the cross-sectional relationship between smoking status, respiratory symptoms and the SF-6D utility scores in the population attending primary care clinic. The population was older age, predominantly men with lower educational level. The preference-based utility score may not be applicable to general population with other characteristics.

## Conclusions

The SF-6D utility scores of subjects attending primary care clinics with a history of smoking were calculated. The SF-6D utility score of current smokers was not significantly different from that of ex-smokers. The SF-6D utility score of subjects had smoked heavily also was not significantly different from those who had smoked moderately or lightly. Women or presence of any respiratory symptom (especially breathlessness) reported significantly lower SF-6D utility scores.

## Abbreviations

BCSS: Breathlessness, cough, sputum scale
BMI: Body Mass Index
C.I.: confidence interval
COPD: Chronic Obstructive Pulmonary Disease
FEV1: Forced Expiratory Volume in one second
FVC: Functional Vital Capacity
GATS: Global Adult Tobacco Survey
GOPC: General Outpatient Clinic
HRQoL: Health-Related Quality of Life
MOS SF-36: Medical Observation Survey Short-Form 36
QALYs: quality-adjusted life years
S.D.: Standard Deviation
SF-12: Short Form 12
SF-6D (HK): Short Form 6 Dimension Hong Kong
